# Next-generation biodegradable cardiovascular stents: integrating biosensors and personalized drug delivery for improved outcomes

**DOI:** 10.1097/MS9.0000000000003941

**Published:** 2025-09-23

**Authors:** Hamza Sajid, Ali Hamza, Maliha Khalid, Muhammad Talha, Aminath Waafira

**Affiliations:** a Department of Medicine, Allama Iqbal Medical College, Lahore; b Department of Medicine, Mayo Hospital, Lahore, Pakistan; c Jinnah Sindh Medical University,Karachi; d Department of medicine, King Edward Medical University, Lahore, Pakistan; e The Maldives National University, Malé, Maldives

**Keywords:** biodegradable stents, biosensors, cardiovascular interventions, personalized medicine

## Abstract

Cardiovascular stents have evolved from bare metal stents to drug-eluting stents, improving outcomes in coronary interventions but posing risks of late thrombosis, inflammation, and long-term safety concerns. Biodegradable stents represent the next generation of innovation, designed to degrade into non-toxic products while minimizing inflammatory responses. Recent advances suggest further potential through the integration of biosensors and personalized drug delivery systems. Biosensors allow real-time monitoring of endothelial healing, inflammatory activity, restenosis, and local drug levels, enabling early detection of complications and timely intervention. Personalized drug release, guided by patient-specific profiles and comorbidities, can optimize therapeutic efficacy while reducing adverse effects. Although biodegradable stents face challenges related to mechanical strength, degradation timing, and implantation complexity, their combination with intelligent biosensing and adjustable drug delivery systems offers a transformative approach to reducing cardiovascular disease morbidity and mortality. The integration of these technologies may redefine future standards in interventional cardiology.

*To the editor,*

Cardiovascular diseases (CVDs), the major cause of increasing morbidity and mortality rates worldwide, are being effectively treated by percutaneous coronary interventions, including stents. Over the past years, the technology of cardiovascular stents has developed significantly from bare metal stents (BMS) to drug-eluting stents (DES). DESs are efficient in reducing the stent thrombosis (ST) and in-stent restenosis (ISR), but they increase the risk of late stent thrombosis (LST), inflammatory reactions, and long-term safety[1]. A study also showed the safety and patency benefits of DESs over BMSs, placing them as the first-line stent for femoropopliteal artery lesions[2]. The development of intelligent cardiovascular stents, which are biodegradable and can provide real-time monitoring capabilities along with personalized drug release profiles based on individual patient characteristics, can resolve these complications.

Biodegradable stents disintegrate into non-toxic products within months to years after the vessel has been healed completely. These may be polyesters, bacterially derived polymers, or corrodible metals, which cause little inflammatory response on degradation and are comparatively safer to use[3]. However, researchers are trying to resolve the problem of inflammation by developing better-quality biodegradable stents. The use of biosensors in biodegradable DES can further increase their effectiveness, as they allow real-time monitoring of endothelial healing, inflammatory response, ISR, and local drug concentration[4]. These sensors can help in early detection of in-stent stenosis and graft failure, providing early treatment to the patient, limiting complications, and reducing the need for complex procedures such as bypass.

Traditional DESs release a fixed amount of drug (either anti-inflammatory or antithrombic) to avoid the risk of restenosis. But in patients with associated comorbidities and genetic variabilities, drug dosage should be adjusted according to the patient’s profile. Using advanced biomaterial stents with biosensors and adjustable polymer coatings, which release specific amounts of drugs based on the patient profile, enhances the precision and accuracy of the treatment and can give better results[5].

These advanced biomaterial stents can give better safety profiles and outcomes when compared to the traditional DESs. A comprehensive analysis comparing biodegradable drug-eluting stents to conventional stents in patients with acute coronary syndrome revealed that biodegradable stents are associated with a slight reduction in the rates of ST, major adverse cardiovascular events, target vessel revascularization, and target lesion revascularization. This supports their effectiveness in treatment[6]. Besides these benefits, the degradation time of these stents is not always compatible with the standard time required to heal the vessel. Moreover, the strength of these biodegradable stents and the difficulty of implantation are major concerns[7].

In conclusion, integrating biodegradable materials, biosensor technology, and personalized drug delivery systems into cardiovascular stents marks a pivotal advancement in interventional cardiology. Ongoing research and development will be essential to optimize these technologies, improve affordability, and enable broader adoption, ultimately helping to reduce CVD-related mortality and enhance patient outcomes. This letter to editor is in compliance with the TITAN guideline[8].

## Data Availability

No data were generated for this manuscript.

## Research registration unique identifying number (UIN)

Name of the registry: NA; Unique Identifying number or registration ID: NA; Hyperlink to your specific registration (must be publicly accessible and will be checked): NA.

## Peer and Provenance Statement

This article was not commissioned and was externally peer-reviewed.

## References

[1] McKavanagh P Zawadowski G Ahmed N. The evolution of coronary stents. Expert Rev Cardiovasc Ther 2018;16:219–28. 29381087 10.1080/14779072.2018.1435274

[2] Gouëffic Y Torsello G Zeller T. Efficacy of a drug-eluting stent versus bare metal stents for symptomatic femoropopliteal peripheral artery disease: primary results of the EMINENT randomized trial. Circulation 2022;146:1564–76. 36254728 10.1161/CIRCULATIONAHA.122.059606

[3] Dinc R Ekingen E. Biodegradable stents in the treatment of arterial stenosis. J Clin Med 2025;14:532. 39860538 10.3390/jcm14020532 PMC11765601

[4] Zhang X Li L Deng Z. Liquid metal-based flexible bioelectrodes for management of in-stent restenosis: potential application. Biosensors (Basel) 2023;13:795. 37622881 10.3390/bios13080795 PMC10452354

[5] Zhang K Liang W Chen X-B. Smart materials strategy for vascular challenges targeting in-stent restenosis: a critical review. Regen Biomater 2025;12:rbaf020. 40290450 10.1093/rb/rbaf020 PMC12034381

[6] Yuan H Wu Z Lu T. Comparison of biodegradable and durable polymer drug-eluting stents in acute coronary syndrome: a meta-analysis. BMJ Open 2022;12:e058075. 10.1136/bmjopen-2021-058075 PMC9185674 35676012

[7] Shen Y Yu X Cui J. Development of biodegradable polymeric stents for the treatment of cardiovascular diseases. Biomolecules 2022;12:1245. 36139086 10.3390/biom12091245 PMC9496387

[8] Premier Science, London, UK, Agha R Mathew G Rashid R. Transparency in the reporting of Artificial INtelligence – the TITAN guideline. PJS. 2025. Accessed 26 May 2025. https://premierscience.com/pjs-25-950/

